# A nomogram for predicting low bone mineral density in the elderly using chest CT

**DOI:** 10.3389/fendo.2026.1818129

**Published:** 2026-05-14

**Authors:** Yali Zou, Runhua Zhou, Di Wu

**Affiliations:** Department of Radiology, The Eighth Affiliated Hospital of Sun Yat-sen University, Shenzhen, China

**Keywords:** bone mineral density, chest computed tomography, nomogram, opportunistic screening, osteoporosis

## Abstract

**Introduction:**

Osteoporosis leads to fragility fractures and imposes a severe health burden on aging populations. Early detection of bone loss is essential for effective clinical intervention. This study aimed to develop a predictive nomogram for low bone mineral density (BMD) by leveraging opportunistic chest CT scans and clinical data.

**Methods:**

This study performed a retrospective analysis of 217 patients, consisting of 71 males and 146 females. All patients underwent chest CT and dual-energy X-ray absorptiometry (DXA) within 30 days. The lowest DXA T-score was used to stratify participants into Normal and Low BMD groups. Univariate and correlation analyses were conducted for all variables, followed by multicollinearity checks. Multivariate logistic regression analysis was then performed to identify key predictors. Model performance was evaluated using ROC curves and validated with bootstrap calibration and Decision Curve Analysis (DCA).

**Results:**

T11 CT attenuation, age, and BMI emerged as significant independent predictors of diminished BMD (all P < 0.05). The final predictive model demonstrated good discriminatory performance, with an AUC of 0.864 (95% CI: 0.813–0.914). Furthermore, the Hosmer-Lemeshow test resulted in a p-value of 0.197, indicating a favorable model fit. DCA and calibration plots confirmed high clinical net benefit and predictive accuracy.

**Discussion:**

This opportunistic CT-based nomogram can effectively identify individuals at risk for bone mass deterioration. It can be used to opportunistically predict the risk of bone loss in middle-aged and older adults, and may serve as a cost-effective screening approach for older populations.

## 1 Introduction

As the global population ages, osteoporosis has become a growing public health concern (1). This metabolic bone disorder leads to around 9 million fragility fractures each year (2). Bone mass reduction, a key precursor to osteoporosis, often occurs without noticeable symptoms. Research shows that the risk of bone mass decline significantly increases starting at age 40 (3). Therefore, monitoring bone health in middle-aged and elderly individuals is essential to prevent the progression to osteoporosis.

Bone mineral density (BMD) measurement is currently the primary method for assessing bone quality, with dual-energy X-ray absorptiometry (DXA) being the gold standard recommended by the WHO (4, 5). This method works by emitting X-rays at two energy levels. The difference in absorption of high- and low-energy X-rays by various tissues is used to calculate bone density. Bone tissue absorbs more low-energy X-rays than soft tissue, and the system uses absorption in bone-free areas as a baseline to estimate bone mineral content by comparing X-ray attenuation (6). However, DXA requires specialized equipment, and differences in technologies or databases across manufacturers can complicate the comparison of results (7).

Recent studies have shown that the tissue linear attenuation coefficient measured in Hounsfield units (HU) from computed tomography (CT) strongly correlates with BMD. Spinal vertebral HU values have been used to identify reduced bone density (8, 9). Thoracic CT, commonly employed for lung cancer screening and pulmonary nodule follow-up, provides thoracic vertebral imaging that can be utilized for BMD assessment (10, 11). However, most studies have focused on the correlation between HU thresholds and BMD, with limited research integrating clinical information with radiographic indicators. While HU values offer some diagnostic value, their sensitivity and specificity can be inconsistent due to the influence of factors such as age, body mass index (BMI), chronic diseases, and medication use on BMD (12).

This study aims to combine thoracic CT imaging features of the thoracic spine with clinical data to develop a BMD prediction model. Additionally, a nomogram will be created and validated to provide a visual tool for assessing BMD.

## 2 Materials and methods

### 2.1 Study population

We conducted a retrospective analysis of medical and radiological data from 421 patients who underwent DXA examinations at our hospital between July 2024 and January 2025. All participants were required to be over 40 years of age and have chest CT imaging data obtained within 30 days of their DXA examination. The exclusion criteria included (1): Incomplete imaging data, such as missing chest CT scans (not including all 12 thoracic vertebrae) or incomplete DXA scan data. (2) Lack of electronic medical records or incomplete documentation. (3) A history of fractures or prior surgery at T11, T12, or the left hip. (4) Severe spinal deformities that hindered the acquisition of standard spinal imaging planes. (5) A history of osteoporosis treatments (such as vitamin D supplementation, denosumab injections).

This study was approved by our hospital’s Ethics Review Committee (Approval No.: 2025-049-01). Given the retrospective nature of the study, the committee waived the requirement for patient informed consent.

### 2.2 Minimum sample size estimation

The minimum sample size was calculated using the events per variable (EPV) method (13). This study aimed to include 3 variables (k). Given the low BMD incidence rate of r = 146/217 = 0.672 and an EPV of 20, the required minimum sample size was calculated as follows:


$$N_{min}=\frac{\left(EPV\times k\right)}{r}=\frac{\left(20\times 3\right)}{0.672}\approx 89$$

### 2.3 Data collection

Baseline data were collected from electronic medical records and included gender, age, BMI, history of diabetes, hyperlipidemia, long-term steroid use (over 3 months), and alcohol abuse.

All DXA data were collected using the Horizon-Wi DXA scanner (Hologic, Inc., Waltham, MA), with daily calibration to ensure accuracy. Trained operators standardized spinal (L1-L4) and left hip positioning, delineated regions of interest (ROIs), and performed automated analysis. Patients were classified into three groups based on the minimum T-score according to World Health Organization criteria: normal (T-score ≥ -1.0), osteopenia (-1.0 > T-score > -2.5), and osteoporosis (T-score ≤ -2.5) [5].

Chest CT data were primarily obtained from the Aquilion Prime TSX-303A (Canon Medical Systems, Otawara, Japan) and uCT 530 (United Imaging Healthcare, Shanghai, China). Scan parameters were standardized with a tube voltage of 120 kVp, automatic tube current, and reconstruction settings of 1 mm slice thickness using a soft tissue window. Patients were instructed to inhale and hold their breath during the scan to capture the chest CT images. Daily calibration was performed on each device to ensure the accuracy of CT Hounsfield units.

This study focused on the 11th and 12th thoracic vertebrae, which are most susceptible to fragility fractures, as the measurement targets (14). All HU measurements were performed using the Picture Archiving and Communication System (PACS). The process followed these steps: Two authors with over five years of experience opened the chest CT images using the Multi-Planar Reconstruction (MPR) tool. Using the ROI tool in the mid-sagittal plane of the thoracic spine, they delineated regions of interest on the T11 and T12 vertebral bodies and extracted the HU values. The ROI delineation method followed the approach described by Ehresman et al. for lumbar MRI measurements (15), focusing on the trabecular bone region of the vertebral body while excluding cortical bone, hemangiomas, venous plexuses, and Schmorl’s nodes. The final HU value was calculated as the mean of the two authors’ measurements.

### 2.4 Statistical analysis

Statistical analysis and graphing were performed using RStudio 2024.12.1 + 563 (RStudio, pBC, Boston, MA, USA). Demographic and radiological data were grouped based on DXA results. Normally distributed variables are presented as mean ± standard deviation, skewed variables as median (interquartile range), and categorical variables as count (percentage). Group differences were analyzed using one-way ANOVA or chi-square tests. Spearman’s correlation coefficient was used to assess associations between variables. Multicollinearity among variables was checked using variance inflation factors (VIF), excluding those with a VIF > 5. Variables with *p* < 0.2 in univariate analysis were included in multivariate logistic regression to identify risk factors for BMD decline and construct the nomogram prediction model. Internal validation of the model was performed using bootstrap resampling (B = 1000) and 10-fold cross-validation, with decision curve analysis (DCA) evaluating clinical utility. All tests were two-tailed, with *p* < 0.05 considered statistically significant. Intraclass correlation coefficients (ICC) were used to assess measurement consistency between two assessors.

## 3 Results

### 3.1 Participant characteristics and HU values

**Figure 1:**
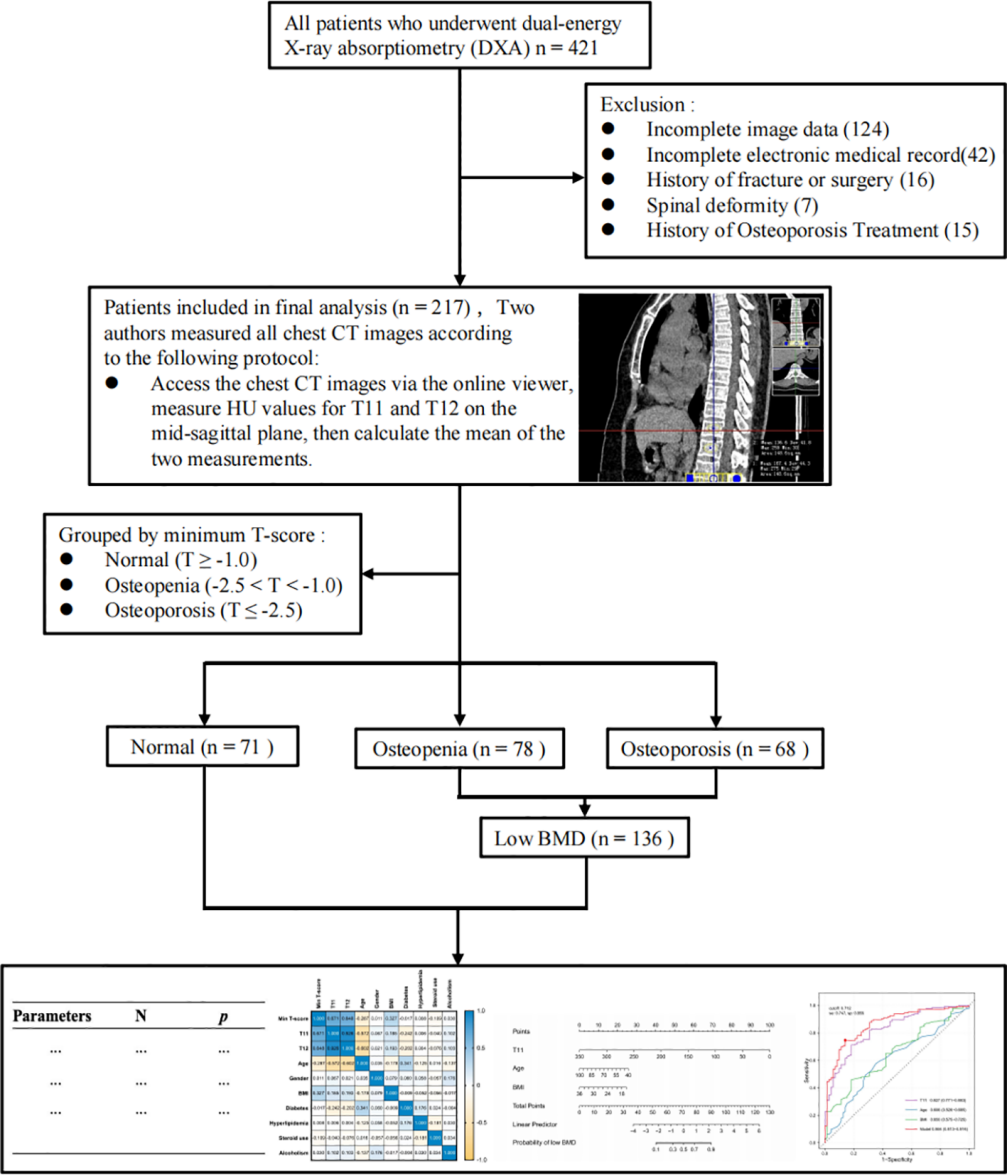
Study flow chart.

This study included 217 patients, all of East Asian ethnicity (**Figure 1**). Based on their minimum T-scores, participants were classified into three groups: normal (n=71), osteopenia (n=78), and osteoporosis (n=68). As bone mass decreased, T11 HU values (*p* < 0.001), T12 HU values (*p* < 0.001), and BMI (*p* < 0.001) progressively declined, while age increased (*p* < 0.001). There was a difference in gender distribution (p = 0.077), with more females than males in all three groups. No significant differences were found for other variables (**Table 1**). The inter-rater intraclass correlation coefficients (ICC) for T11 and T12 measurements were 0.954 (95% CI: 0.941-0.965) and 0.944 (95% CI: 0.928-0.957), respectively, indicating excellent reproducibility (**Supplementary Figure 1**).

**Table 1:** Descriptive analysis of the study populations.

Parameters	Normal (n = 71)	Osteopenia (n = 78)	Osteoporosis (n = 68)	*p*
T11 HU value	224.9 ± 47.0	183.4 ± 44.0	130.6 ± 43.7	**<0.001**
T12 HU value	209.8 ± 46.2	171.6 ± 39.0	123.8 ± 41.9	**<0.001**
Age (y)	54.0 (50.0, 62.5)	55.5 (49.0, 64.5)	63.5 (55.8, 73.2)	**<0.001**
BMI (Kg/m^2)	24.7 (23.0, 27.0)	24.0 (22.4, 26.3)	22.4 (20.9, 24.1)	**<0.001**
Gender, n(%)				0.077
Male	20 (28.2)	33 (42.3)	18 (26.5)	
Female	51 (71.8)	45 (57.7)	50 (73.5)	
Diabetes, n(%)				0.971
Yes	19 (26.8)	20 (25.6)	17 (25.0)	
No	52 (73.2)	58 (74.4)	51 (75.0)	
Hyperlipidemia, n(%)				0.137
Yes	26 (36.6)	40 (51.3)	26 (38.2)	
No	45 (63.4)	38 (48.7)	42 (61.8)	
Steroid use, n(%)				0.120
Yes	3 (4.2)	5 (6.4)	9 (13.2)	
No	68 (95.8)	73 (93.6)	59 (86.8)	
Alcohol abuse, n(%)				1.000
Yes	3 (4.2)	3 (3.8)	2 (2.9)	
No	68 (95.8)	75 (96.2)	66 (97.1)	

Bold values ​​indicate statistically significant differences.

### 3.2 Univariate logistic regression analysis

Univariate analysis identified several risk factors for reduced bone mass, including decreased T11 and T12 HU values, increased BMI-adjusted age, and reduced BMI (*p* < 0.05). Long-term steroid use (OR = 2.404, *p* < 0.2) showed trends toward an increased risk. However, gender, hyperlipidemia and a history of alcohol abuse were not significantly associated with bone mass reduction (**Table 2**).

**Table 2:** Univariate logistic regression analysis for the prediction of low BMD (osteopenia+osteoporosis).

Parameters	Coefficient	AUC (95%CI)	OR (95%CI)	*p*
T11 HU value	-0.028	0.827 (0.771 ~ 0.883)	0.973 (0.964 ~ 0.980)	**<0.001**
T12 HU value	-0.029	0.820 (0.761 ~ 0.879)	0.971 (0.962 ~ 0.979)	**<0.001**
Age	0.033	0.606 (0.526 ~ 0.685)	1.034 (1.007 ~ 1.063)	**0.014**
BMI	-0.152	0.650 (0.575 ~ 0.725)	0.859 (0.784 ~ 0.937)	**<0.001**
Gender (Female)	-0.314	0.534 (0.468 ~ 0.599)	0.730 (0.388 ~ 1.344)	0.320
Diabetes	-0.074	0.507 (0.444 ~ 0.570)	0.929 (0.492 ~ 1.793)	0.823
Hyperlipidemia	0.356	0.543 (0.473 ~ 0.612)	1.428 (0.802 ~ 2.578)	0.231
Steroid use	0.877	0.527 (0.493 ~ 0.560)	2.404 (0.753 ~ 10.685)	0.180
Alcohol abuse	-0.218	0.504 (0.476 - 0.532)	0.804 (0.192 ~ 4.010)	0.769

Bold values ​​indicate statistically significant differences.

### 3.3 Heatmap and multicollinearity analysis

**Figure 2:**
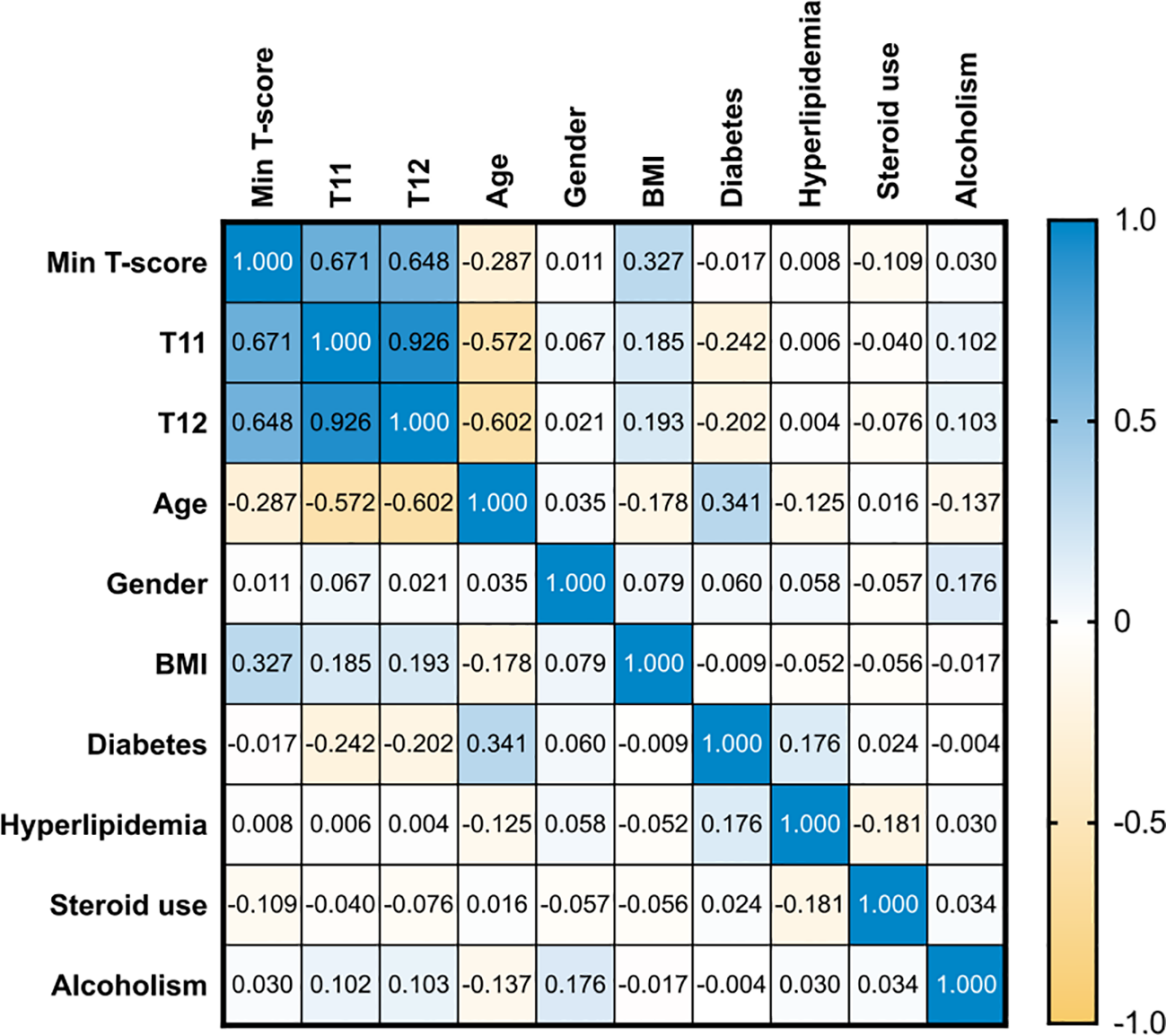
Heatmap of spearman correlation for each parameter.

In the heatmap, blue indicates a positive correlation, while yellow represents a negative correlation, with darker colors reflecting stronger correlations (**Figure 2**). The minimum T-value exhibited moderate positive correlations with T11(r = 0.671) and T12 (r = 0.648), while age showed moderate negative correlations with T11 (r = -0.572) and T12 (r = -0.602). T11 and T12 displayed a strong positive correlation (r = 0.926).

Multiple linear regression analysis was performed on all variables. The results revealed that the VIF for T11 and T12 were 4.354 and 4.490, respectively, significantly higher than those of other variables (VIF ≤1.734). Given the high correlation and moderate multicollinearity, we compared models that included either T11 or T12 while keeping the same set of covariates (age, BMI, gender, diabetes, hyperlipidemia, steroid use, and alcoholism). Nested models were used to assess the complementary gains. The analysis showed that the model including T11 outperformed the model with T12 in terms of information criteria and discriminative power. Adding T12 to the T11 model did not significantly improve the model, whereas adding T11 to the T12 model significantly enhanced the model fit (**Supplementary Table 1**). As a result, T11 was retained, and T12 was excluded.

### 3.4 Multivariate logistic regression analysis

Multivariate logistic regression analysis was conducted using the five factors identified in the univariate analysis as independent variables, with low BMD as the dependent variable. The results indicated that T11 HU (OR = 0.966, 95% CI 0.956–0.976, *p* < 0.001), age (OR = 0.945, 95% CI 0.907–0.983, *p* < 0.01), and BMI (OR = 0.860, 95% CI 0.766–0.959, *p* < 0.01) were independent predictors of low BMD. Although gender was significant in the univariate analysis (p = 0.042), the relatively high standard error (SE = 0.408) suggested instability in the estimate. Given the imbalance in the male-to-female ratio in the sample, gender was excluded from the final model analysis. Steroid use did not reach statistical significance (*p* = 0.212) (**Table 3**).

**Table 3:** Multivariate logistic regression analysis for the prediction of low BMD.

Parameters	*β*	SE	Wald *χ²*	*p*	OR (95%CI)
T11 HU value	-0.034	0.005	44.139	**<0.001**	0.966 (0.956 ~ 0.976)
Age	-0.056	0.021	7.556	**0.006**	0.945 (0.907 ~ 0.983)
BMI	-0.151	0.057	7.015	**0.008**	0.860 (0.766 ~ 0.959)
Gender (Female)	-0.831	0.408	4.154	0.042	0.435 (0.190 ~ 0.949)
Steroid use	1.344	0.903	2.212	2.212	3.832 (0.765 ~ 28.201)

Bold values ​​indicate statistically significant differences.

### 3.5 Evaluating predictive performance of risk factors for low BMD

**Figure 3:**
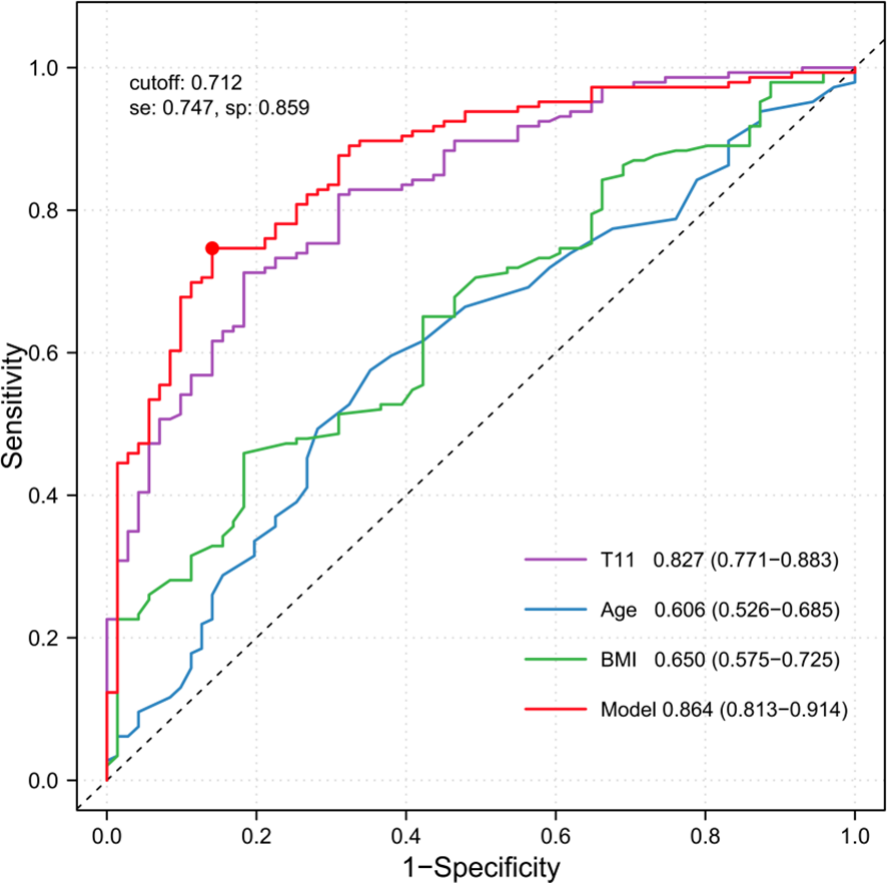
Receiver operating characteristic (ROC) curves for T11, age, BMI and the model.

The ROC curve analysis results showed that the AUC values for predicting low bone mass were 0.827 (95% CI: 0.771–0.883) for T11, 0.606 (95% CI: 0.526–0.685) for age, 0.650 (95% CI: 0.575–0.725) for BMI, and 0.864 (95% CI: 0.813–0.914) for the integrated model (**Figure 3**). Among these, the integrated model demonstrated the best performance, with a diagnostic threshold of 0.712 (sensitivity = 0.747, specificity = 0.859) and significantly superior discriminatory ability compared to T11 (*p* = 0.016).

### 3.6 Development of the prediction model and nomogram

**Figure 4:**
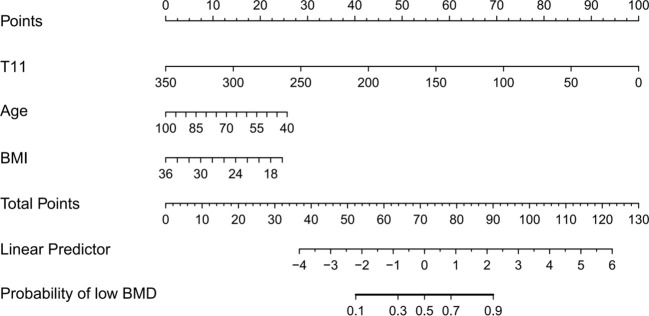
The nomogram of the optimal model for predicting low BMD in middle-aged and older adults. (The points assigned to each variable in the nomogram are summed to obtain a total score, which is then projected onto the risk axis to estimate the probability of low BMD. A higher total score indicates a greater predicted risk).

From the multivariate logistic regression, three significant variables were identified: T11 (X1), age (X2), and BMI (X3). These variables form the following logistic regression equation:logit(P) = 13.494 -0.033*X1 -0.050*X2 -0.143*X3. Based on this model, a nomogram was developed to predict the risk of low bone mineral density (**Figure 4**). The nomogram shows that lower T11 HU values are strongly associated with an increased risk of low bone mass. Additionally, a lower BMI corresponds to a higher risk, while increasing age, when holding T11 and BMI constant, is negatively correlated with the occurrence of low BMD.

### 3.7 Validation of the nomogram

**Figure 5:**
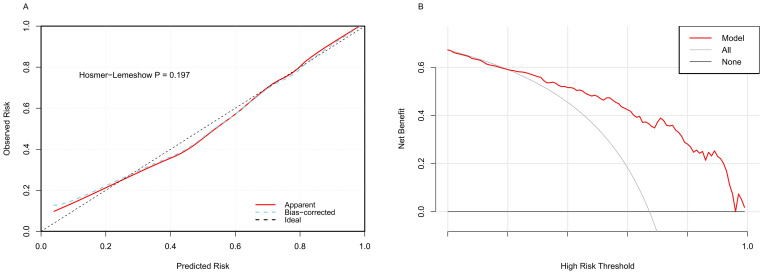
The evaluation of prediction model. **(A)** Calibration curve of the nomogram predicting low BMD in middle-aged and older adults. **(B)** Decision curve of the nomogram predicting low BMD in middle-aged and older adults.

The model was internally validated using a bootstrap method with 1000 resampling iterations. Hosmer–Lemeshow calibration analysis showed a good fit between the model’s predictions and observed values (X² = 11.075, *p* = 0.198). The calibration curve closely matched the ideal reference line (slope = 0.965), indicating that the model’s predicted values for low BMD align well with actual risk (**Figure 5A**). 10-fold cross-validation yielded a mean AUC of 0.860 (SD = 0.028), suggesting stable predictive performance (**Supplementary Figure 2**). DCA demonstrated the nomogram’s strong clinical utility, with a threshold range of 0.01 – 0.92 (net benefit > 0.2) (**Figure 5B**).

## 4 Discussion

Advancing age and BMD are both significant risk factors for fragility fractures (16), highlighting the importance of preventing bone loss in middle-aged and elderly populations. However, despite DXA being the gold standard for bone density measurement, its screening rate among eligible individuals remains below 27% (17). To improve low BMD screening and reduce related fracture risks in these populations, this study used logistic regression analysis to identify three key predictors(T11 HU value, age, and BMI) and developed a nomogram model.

Traditional bone density measurement methods are mainly divided into dual-energy DXA and quantitative computed tomography (QCT) (18). Both require specialized equipment: DXA mandates standardized operation by trained personnel, while QCT relies on a standardized scanning protocol (19, 20). Recent studies have explored using routine clinical CT scans to assess bone density, but relying solely on CT values is prone to confounding factors, leading to poor diagnostic stability (21). This study uses thoracic vertebral imaging data from routine chest CT scans, combined with selected clinical features, to develop a predictive model. This approach enables opportunistic screening for reduced bone mass without additional examination costs or radiation exposure, demonstrating superior diagnostic performance compared to CT value assessment alone. In clinical practice, radiologists can define ROIs in the PACS system to obtain CT values for the T11 vertebra. These values, along with clinical information, are input into the model to calculate the risk. If the predicted probability exceeds the threshold (0.712 in this study), further DXA testing may be recommended for confirmation. In the future, integrating RIS and HIS systems with artificial intelligence could enable automated risk assessment, improving the efficiency of bone mass loss screening.

In this study, T11 HU values showed a significant negative correlation with the occurrence of low BMD in middle-aged and elderly populations (OR = 0.966, 95% CI: 0.956–0.976, *p* < 0.001). This indicates that for every 10 HU increase in T11, the likelihood of low BMD decreases by approximately 41.4%. While previous studies have suggested that T12 has the highest incidence of fragility fractures among vertebrae (22, 23), its diagnostic ability to detect bone mass decline did not exceed that of T11. In univariate analysis, T12’s predictive capability was comparable to T11, with T11 showing a slight advantage. A similar conclusion was reached in Xue et al.’s study (24). This finding suggests that the predictive ability of vertebral HU values for low BMD is not directly linked to vertebral fracture incidence rates and should not be directly extrapolated. Further analysis of collinearity and model comparisons revealed that models incorporating T11, along with variables such as age and BMI, demonstrated better fit and discriminative power than models based on T12. Additionally, T11 is more easily captured in the standard field of view of routine chest CT scans, enhancing its practicality for clinical use.

All HU values were measured using a single elliptical ROI on sagittal images. To minimize potential errors and reproducibility issues associated with single-ROI measurements (25), we fixed the measurement plane to the mid-sagittal plane and assessed intra-rater reliability between two independent assessors. The results showed strong alignment, indicating high reproducibility and reliability of this method. Compared to the traditional axial measurement method, which involves taking three consecutive measurements at different vertebral levels on axial images and averaging the results (26), the single-ROI sagittal measurement simplifies the procedure, improves efficiency, and reduces potential inter-operator variability. Previous studies have shown no significant difference between HU values obtained from sagittal and axial plane measurements (27), further validating the clinical feasibility of the sagittal plane approach.

Notably, in the multivariate analysis, the regression coefficient for the age variable reversed direction, which appears to contradict the commonly held belief that the risk of low BMD increases with age (28). However, there is a common misconception in multivariate regression models: regression coefficients cannot be interpreted as the independent causal effects of each variable on the outcome (29). In this study, the regression model shows that, for the same T11 HU values and BMI, the risk of bone loss is lower with increasing age. Specifically, when BMI is constant, an 80-year-old with the same CT values as a 50-year-old would have better bone quality and, therefore, a lower risk of bone loss. Although there is a moderate negative correlation between age and T11 HU values (r = -0.572), multicollinearity analysis revealed that the VIF for each variable was ≤1.359, ruling out the possibility of multicollinearity between HU values and age.

BMI, calculated based on height and weight, is a widely used indicator in physical screening. Most studies suggest a positive correlation between BMI and BMD (30, 31). However, research by Wang et al. (32) indicates that body weight consists of lean tissue mass (LTM) and fat mass (FM), and the effects of these two components on bone density differ between younger and older individuals. While LTM has shown a stronger correlation with bone mass decline in the elderly (33), measuring LTM requires body composition assessments, which can introduce inter-center variability due to differences in methods and equipment. In contrast, BMI serves as a simpler and more reproducible indicator of body shape, offering easier data acquisition and consistent results across centers. In this study, BMI demonstrated a stable positive correlation with BMD in both univariate and multivariate analyses. As a reliable and easily reproducible surrogate indicator, BMI holds practical value for risk assessment.

This study has several limitations. First, all participants were selected from patients who underwent chest CT scans at the same center, which may limit the generalizability of the sample to the broader middle-aged and elderly population. Additionally, the retrospective nature of the data collection, based on existing medical records, may have led to incomplete or unevenly distributed clinical information. Although predefined inclusion criteria and continuous enrollment were used to minimize bias, selection bias could not be fully eliminated. Second, due to missing data in the medical records, certain known osteoporosis-related factors (such as physical activity, menopausal status, calcium intake, and smoking history) were not included. These factors could act as potential confounders and influence the model’s predictive performance. Third, HU values were measured for only a subset of vertebrae, and the discriminatory ability of other vertebrae was not assessed. Moreover, relevant factors, such as vertebral morphology and paravertebral muscle characteristics, were not included in the analysis. Finally, the predictive model underwent only internal validation, and given the limited sample size, its performance may be overestimated. Prospective multicenter studies with larger sample sizes are needed to validate the model’s robustness and generalizability before it can be applied in clinical practice.

## 5 Conclusions

The nomogram model developed from chest CT scans demonstrates strong discriminatory power, calibration, and practicality. It can be used to opportunistically predict the risk of bone loss in middle-aged and older adults. This model may provide clinicians with a convenient and visual tool for identifying bone loss risk opportunistically, serving as a reference for further testing.

## Data Availability

The raw data supporting the conclusions of this article will be made available by the authors, without undue reservation.
