# Supplementary material for: A nomogram for predicting low bone mineral density in the elderly using chest CT
Source: Front Endocrinol (Lausanne). 2026 May 14;17:1818129. doi: 10.3389/fendo.2026.1818129 (PMC13215837; doi:10.3389/fendo.2026.1818129)
Supplement: Supplementary file 1 [file DataSheet1.docx]

**Table S1** Model comparison based on AIC, BIC, AUC and likelihood ratio tests

| **Parameters** | **Model A** | **Model B** | **Difference / Statistical test** |
| --- | --- | --- | --- |
| AIC | **194.047** | 201.632 | Δ(B–A) = +7.584 |
| BIC | **224.466** | 232.051 | Δ(B–A) = +7.584 |
| AUC (95%CI) | **0.883 (0.836–0.930)** | 0.866 (0.814–0.917) | *p* = 0.234 |
| LRT *p*  (T12 added to Model A) | — | — | 0.251 |
| LRT *p*  (T11 added to Model B) | — | — | **0.003** |

Model A: T11+Age+BMI+Gender+Diabetes+Hyperlipidemia+Steroid+Alcohol abuse, Model B: T12+Age+BMI+Gender+Diabetes+Hyperlipidemia+Steroid+Alcohol abuse.
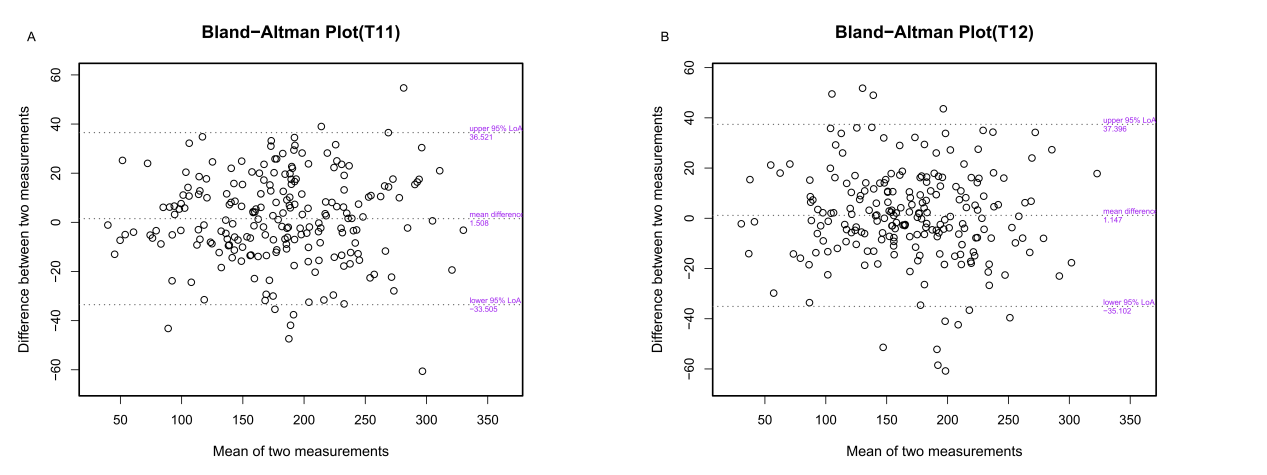


**Figure S1** Bland–Altman plots for agreement between two measurements.(A)T11.(B) T12.

**
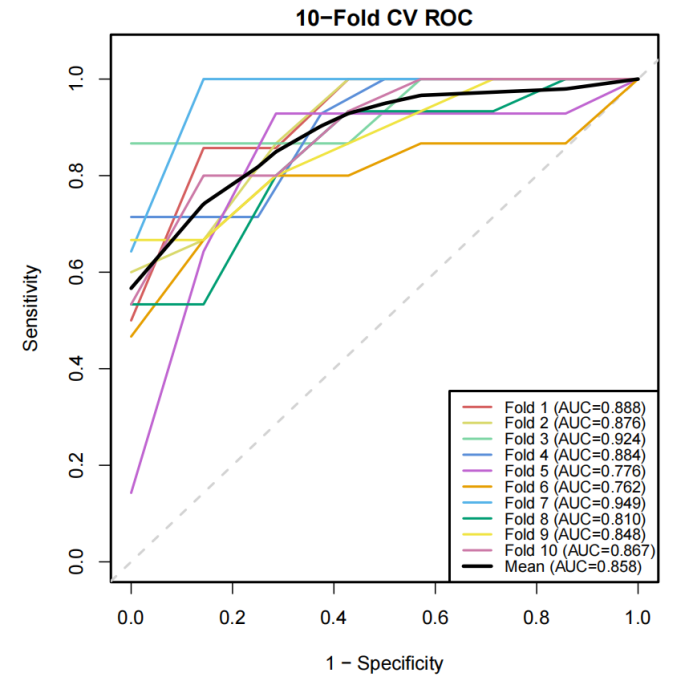
**

**Figure S2 10-Fold Cross-Validation plot** .
